# Seroprevalence and Molecular Epidemiology of Hepatitis B and D Viruses in Mauritania: a Systematic Review and Meta-Analysis

**DOI:** 10.1007/s44197-026-00559-2

**Published:** 2026-04-17

**Authors:** Sidi Mohamed Cheikh Bouna, Nabil Said Abdellah, Habiba Kamal, Mohamed Hemeyine, Ahmed Houmeida, Vanessa Meier Stephenson, Sayeh Ezzikouri, Soo Aleman, Souad Aboudkhil

**Affiliations:** 1 Laboratory of Biotechnology, Agroalimentaire, Materials and Environment, Faculty of Science and Technology Mohammedia, Hassan II University of Casablanca, BP 146, 20650 Mohammedia, Morocco; 2 National Institute of Hepato- Virology (INHV), Nouakchott, Mauritania; 3 Research Team of Virology, Oncology and Biotechnologies of Laboratory of Virology, Oncology, Biosciences, Environment and New Energies, Faculty of Sciences and Techniques Mohammedia, University Hassan II of Casablanca, 28806 Mohammedia, Morocco; 4 Department of Medicine, Karolinska Institutet, Huddinge, Stockholm, Sweden; 5 Department of Infectious Diseases, Karolinska University Hospital, Stockholm, Sweden; 6 Research Unit On Biomarkers in the Mauritanian Population, Faculty of Sciences and Technology, University of Nouakchott, Nouakchott, Mauritania; 7 Department of Medicine, Division of Infectious Diseases, University of Alberta, Edmonton, AB Canada; 8 Li Ka Shing Institute of Virology, University of Alberta, Edmonton, AB Canada; 9 Virology Unit, Viral Hepatitis Laboratory, Institut Pasteur du Maroc, 20360 Casablanca, Morocco

**Keywords:** HBV, HDV, Epidemiology, Prevalence, HBV genotype, Vaccination, Factors associated, Mauritania

## Abstract

Mauritania has a high prevalence of chronic hepatitis B virus (HBV) and hepatitis D virus (HDV), posing a major public health burden. A comprehensive analysis of seroprevalence, transmission dynamics, and molecular epidemiology would help inform strategies for public health interventions. This study systematically reviews the published literature in the field between 1983 and 2024, employing PRISMA 2020 guidelines. Where insufficient data were available for meta-analyses on specific inquiries, summative descriptions were included. Eight studies involving 19,781 participants were included. The pooled HBsAg seroprevalence was 15.2% (95% CI: 12.8%–18.0%; I^2^ = 91.9%; *p* < *0.0001*) by meta-analysis. Among HBV-exposed individuals, male sex, lower education levels, and certain occupations were more commonly observed. Genotype D dominated HBV strains (50%), followed by E (36.2%), A (12%), and G (0.5%). Among 1739 HBsAg-positive individuals from five studies, the seroprevalence of anti-HDV antibodies was 21.2% (95% CI, 14.7%—29.4%; I^2^ = 94.1%; *p* < *0.0001*), with HDV-RNA detectable in 52.0% of cases. Occupation and geographic location were more commonly observed among HDV-infected individuals. HDV-1 was the predominant genotype (89.3%), followed by HDV-5 (10.7%). Given the dual epidemic of HBV and HDV faced by Mauritania, targeted public health strategies will be needed. This systematic review and meta-analysis outlines some of the known epidemiology, both molecular and social, to aid the focus of public health programs.

**Supplementary Information:**

The online version contains supplementary material available at 10.1007/s44197-026-00559-2.

## Introduction

Hepatitis B virus (HBV) is a hepatotropic DNA virus of the *Hepadnaviridae* family [1]. Despite four decades of universal HBV vaccination efforts, the World Health Organization (WHO) estimates 254 million people remain chronically infected worldwide, with the highest burden of disease in Africa and Asia [2]. Chronic infection with HBV puts individuals at increased risk of complications, including liver fibrosis, cirrhosis, and hepatocellular carcinoma (HCC) [3, 4]. Hepatitis D virus (HDV) is a satellite virus that requires HBV to complete its replication cycle and is therefore only found in those already infected with HBV, with estimates of about 5% of the HBV population co-infected with HDV [5, 6]. HBV-HDV co-infection rapidly increases the progression of the viral complications of disease.

Collectively, chronic viral hepatitis accounts for 820,000 deaths annually, again, with the greatest burden disproportionately affecting low- and middle-income countries (LMICs) [4]. In 2016, the WHO set ambitious targets to eliminate HBV as a public health threat by 2030 [7]. However, current projections indicate that most countries, including Mauritania, are unlikely to meet these targets [8]. Access to care remains critically limited. Fewer than 10% of affected individuals have access to screening, and an even smaller proportion receive treatment. In addition, coverage of the birth-dose vaccination— the main strategy to prevent mother-to-child transmission— remains low at 39% [9]. Although vaccination is a key strategy for preventing new infections and provides protection against both HBV and HDV, access is still not universal [6].

Mauritania is a Northwestern African country with a population of ~ 5 million, as of 2023 [10], where 1.6 million live in its capital, Nouakchott[11]. Mauritania bears one of the highest HBV and HDV prevalence in Africa [12], however HDV remains understudied due to low awareness and underdiagnosis. Despite national efforts to combat viral hepatitis, Mauritania is unlikely to achieve WHO 2030 targets [8], underscoring the need for reassessing whether current public health strategies are sufficient or might benefit from additional approaches. Investment in any new strategies would need to be informed by updated knowledge on the epidemiological burden of viral hepatitis B and D. We aimed therefore to conduct this comprehensive systematic review and meta-analysis to assess the seroprevalence, factors associated with HBV and HDV infections, birth-dose HBV vaccination coverage, and molecular epidemiology of these viruses in Mauritania.

## Materials and Methods

This systematic review and meta-analysis followed the criteria of the PRISMA statement guidelines [13].

### Search Strategy and Study Selection

#### Literature search

A comprehensive search was conducted across Google Scholar, Scopus, PubMed, and Web of science for studies published between 1983 and 2024, using predefined search terms (Supplementary Table 1). Using PubMed, the strategy included the Medical Subject Heading (MeSH) search terms “Mauritania” or “Nouakchott” with either or both of “hepatitis B” and “hepatits D” search terms; for all others, the platforms search terms “Mauritania”, “Nouakchott”, “hepatitis B” and “hepatits D” were similarly used, performing combination searches for each, as described. The references of included articles were manually screened to identify additional relevant publications.

#### Study Selection and Definitions

For inclusion, we required the following: (1) Studies showed number of participants with HBV, and/or HDV infection; (2) HBV infection was defined by the presence of hepatitis B surface antigen (HBsAg); (3) HDV co-infection was defined by detection anti-HDV antibodies or detected HDV RNA replication; (4) articles published in English and French were eligible; (5) only peer-reviewed studies were included and in case of eligible study with missing data, authors were personally contacted.

Information on factors associated with HBV and HDV, as well as serotypes and genotypes, was extracted from some of the studies included in this meta-analysis. Vaccination coverage data were obtained from publicly available sources provided by the WHO through its UNICEF partners.

Exclusion criteria were reviews, short communications, or studies with incomplete or unclear prevalence data.

#### Data Extraction

Two independent reviewers (SCB and NS) screened titles and abstracts, with discrepancies resolved by a third reviewer (SE). In case of an eligible study with missing data, authors were personally contacted.

Data extracted from full-text articles included author, publication year, sample size, population type (general or high-risk), demographic characteristics (age, sex), HBsAg-positive and anti-HDV-positive counts, and study location.

### Quality Assessment

The methodological quality and risk of bias of included studies were independently evaluated by three authors (SCB, NS) using the Joanna Briggs Institute Critical (JBI) Appraisal Checklist for Studies Reporting Prevalence Data [14]. This tool assessed key methodological aspects, including sample representativeness, recruitment methods, data collection, and statistical analysis. Each study was assessed based on the checklist criteria, and discrepancies were resolved through discussion. However, we decided a priori to include all eligible studies regardless of their quality rating.

### Statistical Analysis

Statistical analysis was performed in the RStudio environment using R version 4.4.2 and the *meta* package (version 8.0.2). Proportions were transformed using the logit transformation of proportions (PLOGIT) prior to pooling. Heterogeneity statistics, including τ^2^ (tau-squared), I^2^, and H, were estimated to assess variability across studies [15]. Given the anticipated high degree of heterogeneity across studies, pooled prevalence estimates were calculated using a random-effects model. Due to extreme heterogeneity (I^2^ > 90%) and insufficient data within subgroups, subgroup analysis was not performed. Data on factors associated with HBV and HDV, as well as serotypes and genotypes, were descriptively synthesized from the original studies. Only unadjusted estimates are presented, as none of the included studies provided multivariable-adjusted estimates suitable for meta-regression.

### Ethics Statement

As the study analyzed aggregated and anonymized data from published literature; ethical approval requirement was waived.

## Results

### Identification and Selection of Studies

**Fig. 1:**
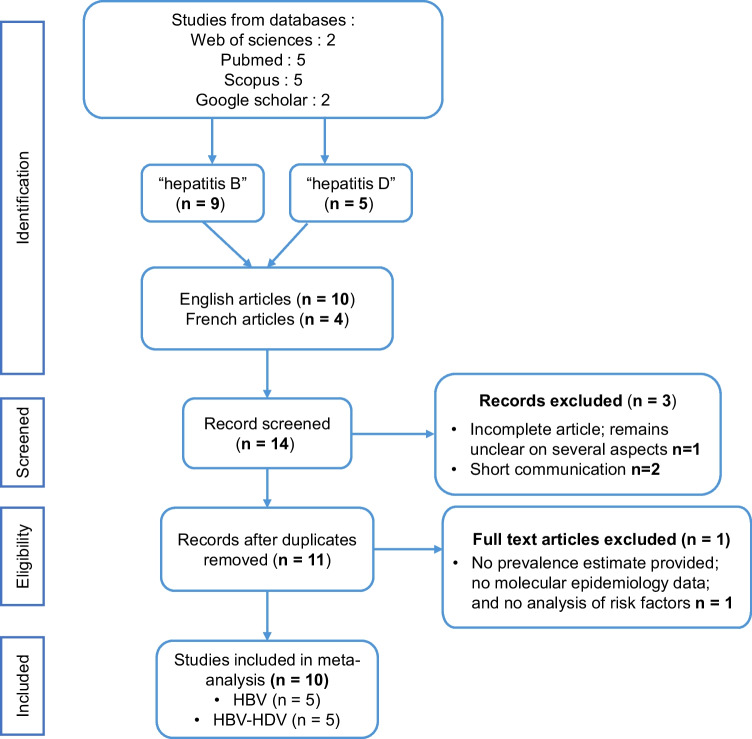
Flow diagram of the screening and selection processes for the meta-analysis of the seroprevalence and molecular epidemiology of hepatitis B and D viruses in Mauritania 1983–2024. †Refers to search strategy with either Mauritania or Nouakchott with either of hepatitis B or hepatitis D search terms. See text and Supplemental Table 1

The PRISMA flowchart outlining the study identification and selection process is shown (Fig. 1). A comprehensive literature search was conducted across PubMed, Google Scholar, Scopus, and Web of Science, yielding 14 relevant publications.

After exclusions, an initial screening based on title and abstract narrowed the selection to 11 articles for full-text review. Following this stage 10 studies met the predefined inclusion criteria and were included in the systematic review and meta-analysis.

### Quality Assessment and Risk of Bias of Included Studies

Among the 10 studies included and assessed for methodological quality using the JBI Critical Appraisal Checklist for Studies Reporting Prevalence Data, 6 (60%) were classified as having a low risk of bias, 3 (30%) as having a moderate risk, and 1 (10%) as having a high risk. JBI quality scores ranged from 3 to 6 out of 9, with a mean score of 4.7 and a median of 5. The JBI items presenting the highest risk of bias mainly concerned the sampling frame, participant recruitment methods, coverage of the analyzed data, and management of non-respondents.

### Epidemiology of HBV in Mauritania

**Fig. 2:**
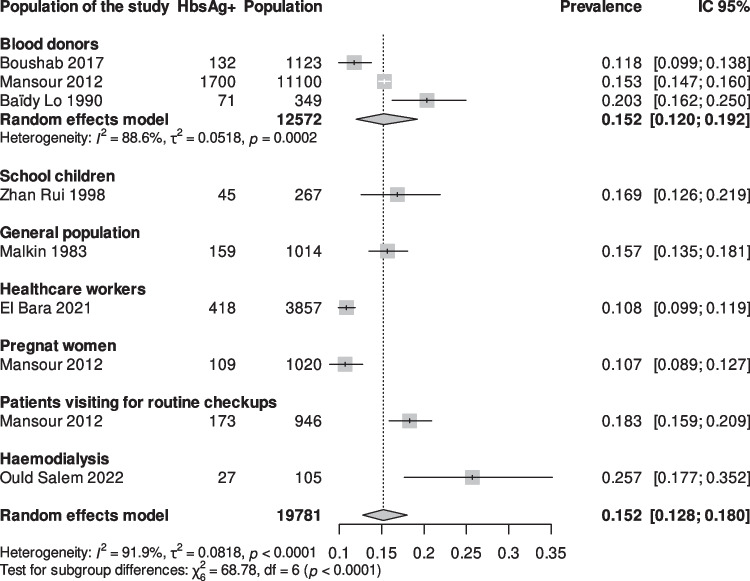
Forest plots of hepatitis B virus infection prevalence in Mauritania 1983–2024

**Fig. 3:**
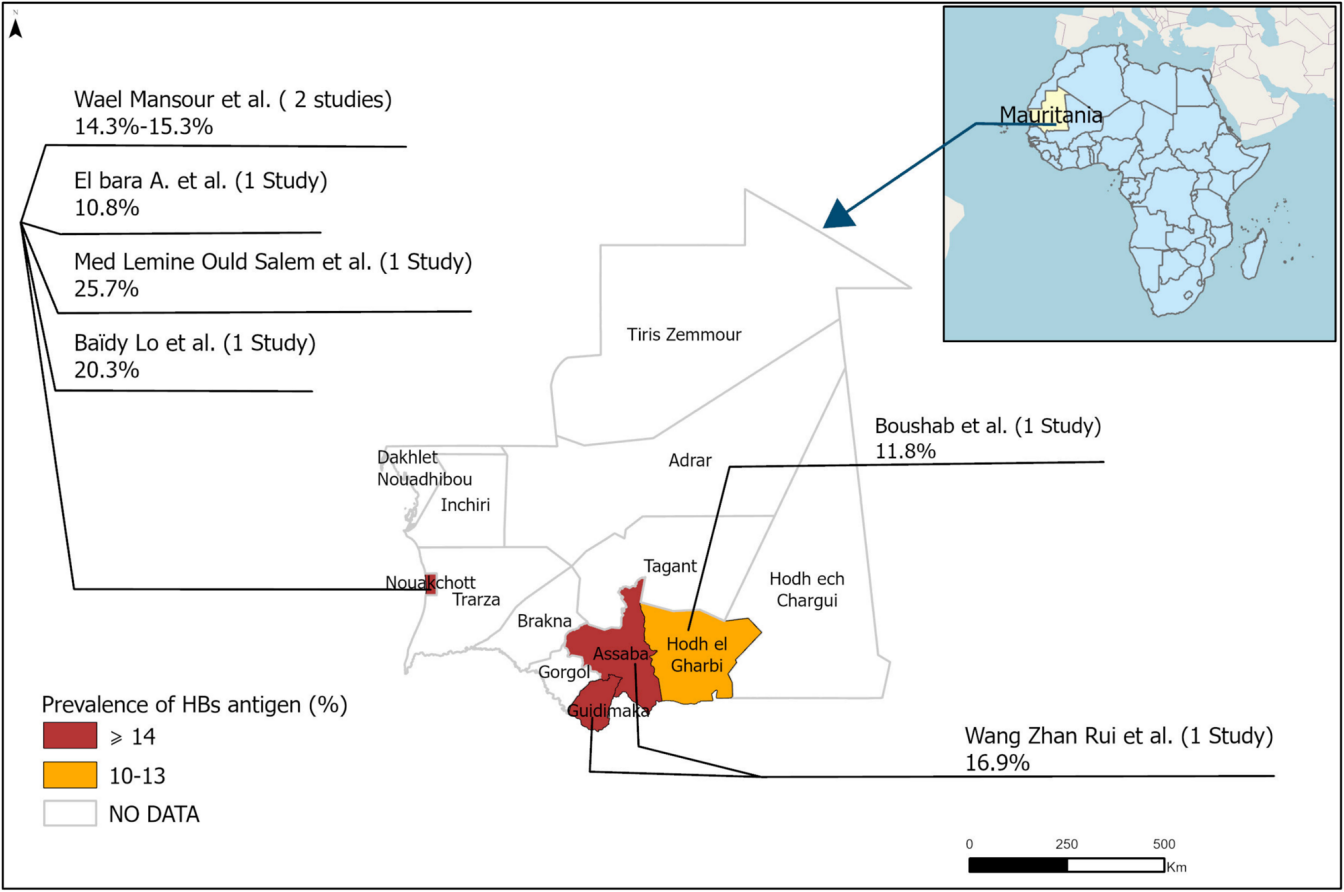
Map of Mauritania showing the distribution of HBsAg prevalence across the country based on data from available studies. Areas in red indicate a prevalence ≥ 14.0%, areas in yellow indicate a prevalence between 10.0% and 13.9%, and areas in white represent regions where no studies have been conducted to date. Data points represent study locations and do not necessarily reflect regional endemicity

A total of 19,781 subjects from 8 studies [16–23] from diverse populations school children [16], pregnant women [21], the general population [18], hemodialysis patients [20], healthcare workers [17] and blood donors [19, 22, 23] were analyzed. Among these, 2834 were HBsAg carriers, yielding an overall HBV prevalence of 15.2% (95% CI: 12.8%–18.0%; *I*^*2*^ = 91.9%; *p* < *0.0001*) (Fig. 2; Table 1). As well, results are displayed by region in Mauritania (Fig. 3).

**Table 1:** Characteristics of the studies included in the review, classified by year of publication

Study ID	Population	Sample size	Age range (years)	HBsAg positive (n)	HBV prevalence estimates %	Anti-HDV Ab (n)	HDV prevalence estimates %	Prevalence of anti-HBc (%)	City	Quality Score	Sex distribution F:M
Wang Zhan Rui et all,. 1998 [16]	**School children**	267	≤ 18	45	16.9	NA	NA	50.2	Kiffa and Selibaby	4/9	10: 35
J.E. Malkin et all,. 1983 [18]	**General Population**	1014	6–22	159	15.7	NA	NA	NA	Atar, Boutillimit, Nema, Lexeibe, Moungual, Thekan, Bouly and Kankoussa	4/9	NA
Wael Mansour et all,. 2012a [21]		946	14–76	173	18.3	31	19.1	76.5	Nouakchott	5/9	75: 98
Boushab Mohamed Boushab et all,. **2017** [19]	**Blood donors**	1123	17–73	132	11.8	NA	NA	NA	Aioun	3/9	NA
Baïdy Lo et all,. 1999 [23]		349	NA	71	20.3	NA	NA	NA	Nouakchott	6/9	0: 71
Wael Mansour et all,. 2012b [22]		11,100	18–65	1700	15.3	447 accepted to participate, 90	20.1	NA	Nouakchott	5/9	15: 342
Wael Mansour et all., 2012a [21]	**Pregnant Women**	1020	14–47	109	10.7	16	14.7	66.3	Nouakchott	5/9	109: 0
Mohamed Lemine Ould Salem et all., 2022 [20]	**Hemodialysis**	105	30–65	27	25.7	NA	NA	NA	Nouakchott	4/9	14: 11
Ahmed El Bara et all., 2021 [17]	**Healthcare workers**	3857	18–59	418	10.8	48	11.5	64.7	Nouakchott	6/9	171: 247
Hélène Le Guillou-Guillemette et all., 2024 [25]	**Patients living with HIV/HBV infection**	292	19–64	292	NA	108	37.0	NA	Nouakchott	5/9	49: 59
Françoise Lunel-Fabiani et all., 2013 [24]	**Patients from hepatology clinics**	300	14–73	300	NA	98	32.7	NA	Nouakchott	5/9	33: 65

Abbreviations: *HBsAg* Hepatitis B surface antigen, *HBV* Hepatitis B virus, *Anti-HDV Ab* Antibodies against Hepatitis D virus, *HDV* Hepatitis D virus, *Anti-HBc* Antibodies against Hepatitis B core antigen, *F* Female, *M* Male, *NA* Not applicable

#### Prevalence of HBV by Different Populations

In three studies including blood donors [19, 22, 23] the pooled prevalence of HBV infection as determined through the meta-analyses was 15.2% (95% CI: 12.0%–19.2%; I^2^ = 88.6%; Fig. 2). The prevalence was 16.9% among school children [16],while lower prevalence was observed among healthcare workers 10.8% [9.9–11.9%] [17] and pregnant women 10.7% [8.9–12.7%] [21]. A higher prevalence was reported in the general population (one study [18]) 15.7% [13.5–18.1%] and in patients undergoing routine health checkups 18.3% [15.9–20.9%] [21], while the highest prevalence was found among hemodialysis patients at 25.7% [17.7–35.2%] [20].

Seven studies [16–22] provided age range data, with an overall mean age of 37.3 years, while HBV showed higher prevalence among men (74.5%) in five studies [16, 17, 21–23].

#### Factors Associated with HBV Infection in Mauritania

Exposure to HBV varied across different socio-demographic and behavioral characteristics reported in the reviewed studies, including sex, education level, ethnicity, history of blood transfusion, and occupation. As no adjusted or pooled analyses were performed, the following findings represent descriptive associations derived from the included studies and should be considered hypothesis-generating rather than causal.

Across several studies, men represented a higher proportion of participants; in studies with a balanced sex distribution, men were observed to have a higher prevalence of HBV compared with women (Table 1) [17–21]. In the pregnancy cohort, among women exposed to HBV, a greater proportion had lower educational attainment, with only 23.4% having completed secondary education [21]. Differences in HBV exposure were also observed across ethnic groups. For example, among healthcare workers, individuals identified as Pulaar/Fula showed lower HBV exposure compared with other ethnic groups [17]. Additionally, some studies reported variations in HBV exposure according to history of blood transfusion and occupation. For instance, students—particularly those enrolled in medical and nursing programs—were observed to have a lower prevalence of HBV compared with non-student workers [17].

#### Genotypes and Serotypes of HBV Infection in Mauritania

Five of the ten studies incorporated data on either serotype or genotype enabling some description here. One study of 1014 individuals from eight villages identified three primary HBV serotypes: ayw2 (34.7%), ayw4 (63.0%), and adw2 (2.3%). The ayw2 serotype was most common in northern regions, while ayw4 dominated in the south. Although no link was found between serotype and ethnicity, geographical differences were significant [18].

Four studies [21, 22, 24, 25] involving 433 patients revealed the following genotype distribution: Genotype D (50.0%), genotype E (36.2%), genotype A (12.0%), and genotype G (0.5%). Notably, 25.0% of genotype D cases belonged to the sub-genotype D8. These findings are summarized in Table 2.

**Table 2:** Distribution of HBV genotypes in the populations studied in Mauritania

HBV genotype	%	Number of individuals	References
D	48.9	45	[22]
E	35.6		
A	15.6		
D	53.0	81	[21]
E	35.0		
A	12.0		
D	42.5	193	[25]
E	38.0		
A	15.5		
G	0.5		
D	60.5	114	[24]
E	34.2		
A	4.3		
G	0.8		
Overall prevalence			
D	49.9	216	[21, 22, 24, 25]
E	36.2	157	
A	11.9	52	
G	0.4	2	

Abbreviations: *HBV* Hepatitis B virus, *HIV* Human Immunodeficiency Virus, *Anti-HDV Ab* Anti-Hepatitis D Virus Antibodies, *HDV* Hepatitis D Virus, *Anti-HBc*, Antibodies against Hepatitis B Core Antigen, *NA* Not available

### Epidemiology of HDV in Mauritania

#### Anti-HDV Positive Prevalence in Mauritania

**Fig. 4:**
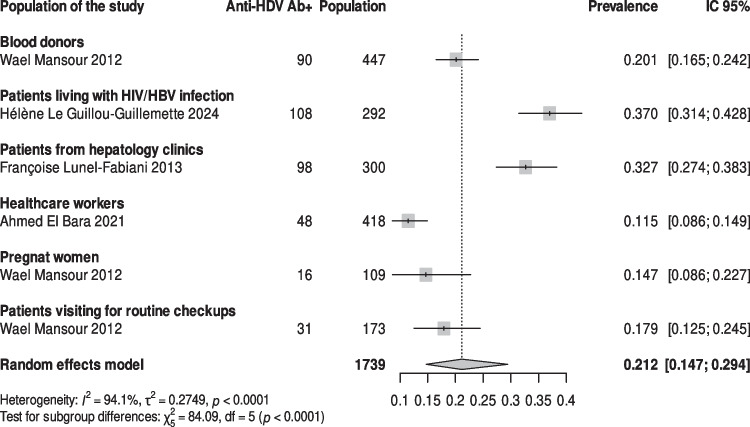
Forest plots of hepatitis D virus seroprevalence among HBsAg-positive patients in Mauritania 2012–2024

All the studies reporting data on HDV prevalence were conducted in the capital Nouakchott [17, 21, 22, 25]. Among 1739 HBsAg-positive individuals, 21.2% (95% CI, 14.7%—29.4%; *I*^*2*^ = 94.1%; *p* < *0.0001*) tested positive for anti-HDV (Fig. 4). The prevalence rates of HDV varied across different populations, reaching 20.1% in blood donors [22], 37.0% in HIV/HBV co-infected patients [25], 11.5% among healthcare workers [17], 14.7% in pregnant women [21] and 32.7% among patients from hepatology clinics [24].

#### Factors Associated with HDV Infection

The presence of hepatitis D virus (HDV) infection in Mauritanians was observed in relation to various sociodemographic, occupational, geographic, and clinical characteristics [21, 22]. Among HBV/HDV co-infected patients, older age was more commonly observed compared with HBV mono-infected individuals 21,22]. Clinically, HDV co-infection was noted alongside more frequent reports of hepatomegaly, hepatic decompensation, and portal hypertension [24].

Descriptive analysis of occupational groups indicated that military personnel showed a higher proportion of HDV seropositivity (33.7%) relative to other occupational categories, including civil servants, merchants, farmers, and artisans (16.3%) [22]. Similarly, geographic observations suggested a higher HDV prevalence in desert regions (26.8%) compared with coastal areas (14.5%) [22].

#### HDV RNA Detection and HDV Genotypes

HDV RNA was tested in 391 anti-HDV positive individuals across the included studies [17, 21, 22, 24, 25]. Viral RNA was detected in 203 of 391 individuals, representing 52.0% (95% CI: 47.0%–56.9%) of tested case based on study-level data. Detection rates varied notably between subgroups, with healthcare workers showing 20.8% and blood donors 62.2% RNA positivity.

Genotypic analysis of 149 cases (Table 3) revealed a predominance of genotype 1 in Nouakchott (89.3% of published cases), followed by genotype 5 (10.7%), highlighting a limited genetic diversity in Mauritania, with no observed differences in disease severity between genotypes [24].

**Table 3:** Distribution of HDV genotypes in the populations studied in Mauritania: Summary of results from published studies

HDV genotype	%	Number of individuals	References
HDV-1	90.3	31	[21]
HDV-5	9.7		
HDV-1	89.3	28	[22]
HDV-5	10.7		
HDV-1	93.0	43	[25]
HDV-5	7.0		
HDV-1	85.0	47	[24]
HDV-5	15.0		
Overall prevalence HDV-1	89.3	133	[21, 22, 24, 25]
Overall prevalence HDV-5	10.7	16	

Abbreviations: *HDV-1* genotype 1 of the Hepatitis D virus, *HDV-5* genotype 5 of the Hepatitis D virus

## Vaccination

As HBV vaccination is crucial to reduce neonatal and early childhood infection, we wanted to assess the prevalence of birth-dose HBV vaccination in Mauritania; however, we did not find any published articles on this subject meeting our inclusion and exclusion criteria. Official statistics for the 3-dose vaccination series coverage from birth-doses from 2005–2024 published online by WHO via its Unicef partners are shown (Supplemental Fig. 1). This data demonstrates that over the last five years vaccination coverage ranged from 85.0% to 97%, peaking in 2020–2021, with an apparent decline in 2022–2024 [26].

## Discussion

In this comprehensive systematic review and meta-analysis spanning publications from 1983 to 2024, incorporating 20,373 individuals across 13 cities in Mauritania, the overall prevalence of HBV infection in Mauritania was 15.2% [95% CI 12.0% – 18.0%]. The highest prevalence observed was among hemodialysis patients (25.7%), while the lowest prevalence was among health care workers (10.8%). HDV infection prevalence is approximately 21.2% [95% CI 14.7% – 29.4%]. This hyper-endemic prevalence of HBV infection is approximately four times higher than the average global prevalence of 3.5% [27], whereas the prevalence of HDV is nearly five times higher than the average global prevalence of 4.5% [28] positioning Mauritania among the countries most affected by HBV and HDV in Africa. In comparison, pooled estimates from recent systematic reviews indicate that HBsAg prevalence in Morocco is around 1.33% (95% CI: 1.07–1.61) [29] in the general population, reflecting a low endemicity level relative to Mauritania’s high rates. Similarly, studies in Tunisia report national HBsAg prevalence around 1.7% in population-based surveys [30], while other Maghreb countries show moderate endemicity levels, with historical estimates ranging between 1.8% and 4.9% across the region [31]. The high endemicity of HBV and HDV infection highlights a critical public health challenge and underscores the need for stronger national prevention, screening, surveillance, and linkage-to-care programs.

This prevalence of HDV is similar to those observed in other high endemic regions of the world. For instance, in West Africa, Cameroon reports a prevalence of 14%, whereas in Central Africa, the Central African Republic shows a 26% prevalence in the general population, reaching 38% among patients with liver diseases. Meanwhile in Gabon, 17% of pregnant women had HDV infection, slightly higher than 14.7% in Mauritania [32].

There is a discrepancy between the high prevalence of HDV in Mauritania and the very low prevalence observed in neighboring countries, notably Mali and Senegal, with estimated rates of 2.7% and 1.4%, respectively [33, 34], despite high HBV endemicity in these countries [35, 36]. Further investigations are needed to determine whether this difference is related to lower HDV screening, sampling bias (e.g., screening of only lower-risk populations), or cultural, social, or host susceptibility factors that could explain these variations. Based on the methods used in the studies, all anti-HDV screenings were performed by ELISA, followed by molecular confirmation with PCR. However, the Malian study used dried blood spot (DBS) samples, while the Mauritanian and Senegalese studies analyzed plasma or serum, which may reduce overall detection sensitivity [37] in the Malian study and contribute to underestimation of prevalence. These methodological differences therefore limit the direct comparability of HDV prevalence between Mauritania and Mali.

Vertical transmission remains the most common route of transmission for HBV worldwide and an important route for maintenance of the viral reservoir in a population [38].The rate of acquisition of chronic HBV is inversely proportional to age, whereby a neonate has over a 90% chance of developing chronic HBV with an exposure as opposed to an adult, which has only about a 5% chance. Additionally, unsafe medical practices, including syringe reuse during mass injection campaigns such as during the early twentieth century to combat diseases like sleeping sickness, may have put Mauritanian residents at increased risk of horizontal transmission [39]-[40]. Understanding these historical practices provides context for the current epidemiological landscape.

In Mauritania, pregnant women are routinely screened for HBV, and when indicated, they receive free antiviral treatment. Although these programs are being implemented, population coverage remains incomplete, and the COVID-19 pandemic has further challenged vaccination efforts, highlighting the need to improve access and expand coverage among at-risk groups [41]. Since 2005 [42], birth-dose HBV vaccination has been implemented. In addition, high-risk newborns receive HBV immunoglobulins within 24 h of birth. Vaccination against HBV, ideally administered at birth, is well established as protective and helps safeguard newborns during this highly vulnerable period, while continuing to confer protection into adulthood, when horizontal exposures become more relevant. This effectiveness is supported by the WHO, which recommends birth-dose vaccination and appropriate antiviral treatment for mothers in its Global Hepatitis Report, emphasizing their critical role in preventing perinatal transmission [43].

Implementation of programs is further complicated by Mauritania’s geography and diverse population. The country spans 1,030,700 km^2^ on Africa’s Atlantic coast and forms a geographic and cultural transition zone between the Maghreb and sub-Saharan Africa.

Furthermore, to ensure the effectiveness of these interventions, a survey of hepatitis B seroprevalence in children is needed to monitor progress in mother-to-child transmission (MTCT) of HBV. In order to strengthen hepatitis B elimination programs, it is important to consider setting up databases in Mauritania to monitor several key indicators, such as the number and percentage of prenatal HBsAg screenings, the number of pregnant women testing positive for HBsAg, the number of HBsAg-positive pregnant women eligible for antiviral treatment, and the number and percentage of these women who actually received antiviral treatment. In addition, it would be useful to track the number and percentage of newborns who receive hepatitis B immunoglobulin at birth.

Male sex accounted for a higher proportion of HBV infections in our dataset, which is consistent with observations from other endemic regions [44]. Similarly, HDV co-infection appeared more common in males (73%) than in females (27%), which may reflect sex-specific differences in risk behaviors or exposures. These observations indicate that gender-sensitive strategies could be useful for promoting awareness and prevention of hepatitis B and D, while remaining descriptive and exploratory.

Among the ten analyzed studies, only five utilized Sanger sequencing methods, and none employed next-generation or whole-genome sequencing. This provides preliminary information on predominant strains within individuals, but may not capture the full diversity of circulating HBV strains. Consequently, the observed distribution of genotypes in Mauritania should be considered descriptive and exploratory, and may generate hypotheses for future studies rather than representing definitive patterns of HBV genetic diversity.

Moreover, current data rely on PCR targeting a single gene, limiting the comprehensive understanding of HBV genotypes or subtypes in Mauritania, or that of circulating strains bearing mutations that predict drug resistance or high-risk of cancer. For the studies that investigated HDV prevalence, ELISA was used for primary detection of anti-HDV antibodies, followed by viral load quantification and Sanger sequencing for genotyping, if positive. Diagnostic limitations persist in Mauritania, where public healthcare infrastructure lacks access to anti-HDV testing or HDV RNA detection, complicating clinical management. Furthermore, the lack of data from most regions hampers the comprehensive comparison of prevalence. Data from underrepresented regions is critical to optimize public health response.

The findings of our review have clear public health and policy implications for Mauritania. The high burden of HBV and substantial HDV co-infection support strengthening the national hepatitis response through improved monitoring and expansion of existing prevention strategies, routine anti-HDV testing among HBsAg-positive individuals, particulary those with advanced liver disease or HIV/HBV co-infection, and stronger linkage to care. The concentration of available data in Nouakchott also highlights the need for robust national surveillance, including data from underserved rural and remote rural regions, to better guide resource allocation and hepatitis elimination efforts.

Our results also suggest a higher prevalence of HDV among high-risk groups, including HIV-infected patients, consistent with published meta-analyses, and among military personnel [22]. As most studies focused on selected populations—such as blood donors, pregnant women, patients undergoing routine check-ups, and healthcare workers—conclusions drawn may not accurately reflect the prevalence in the general population.

Our study has several limitations. The pooled prevalence estimates (15.2% HBV, 21.2% HDV) combine general and high-risk populations, and together with the high heterogeneity (I^2^ = 91.9%), this limits the reliability and national representativeness of our findings. The absence of complete HBV sequences and the limited number of HDV sequences available in the databases limits our ability to do deeper genetic analyses. Most of the included studies were based on specific populations (hospitals, blood donors, schoolchildren, patients followed in a single center), often without randomization or statistical justification. Moreover, response rates and the handling of non-respondents were rarely reported, thereby reducing confidence in the prevalence estimates and limiting their generalizability to the overall Mauritanian population. The majority of the data (80%) originated from Nouakchott, the capital city, yet we sought to present the geographical distribution of HBV prevalence in four of the most populated wilayas. However, the results of the included studies restricted the presentation of data in eight other cities due to imprecise estimates. Consequently, only the overall prevalence reported in the article was retained [18], making a detailed analysis of the virus’s geographical distribution among these regions infeasible. This concentration of data in Nouakchott represents a geographical bias, limiting the representativeness of our findings for rural areas and less-populated regions of Mauritania. Additionally, due to the limited number of included studies, sensitivity analyses were not performed. Although all identified published studies were included, a formal assessment of publication bias was not conducted, and this risk is therefore acknowledged as a limitation of the study. Our estimates should be considered preliminary until more data become available to address the lack of information in the rest of the country and achieve more nationally representative results.

Furthermore, our synthesis of factors associated with HBV and HDV infections is based on descriptive, unadjusted estimates from individual studies, as no multivariable-adjusted estimates were available for meta-regression. This limits our ability to assess independent associations or control for potential confounding.

A third of the examined studies focused on blood donors. In Mauritania, blood donation practices are voluntary and are largely family-based. Thus, this can bias the data to families or communities around an individual requiring care, giving surges of donations from single communities who may have similar exposure risks. Although the current HBV screening protocols at the National Blood Transfusion Centre utilize rapid tests to detect HBsAg, followed by ELISA tests for confirmation. The lack of nucleic acid amplification testing poses a risk of missing occult infections.

## Conclusion

This systematic review and meta-analysis shows that HBV and HDV remain important public health challenges in Mauritania. Important gaps remain regarding rural populations, occult HBV, HBV- and HDV-associated hepatocellular carcinoma, and factors associated with these infections. The findings support strengthening screening, linkage to care, and monitoring of existing prevention programs, particularly antenatal HBsAg screening, timely birth-dose vaccination coverage, follow-up of exposed infants, and targeted HDV testing among HBsAg-positive individuals. They also highlight the need for stronger surveillance systems and more nationally representative data to identify implementation gaps, guide resource allocation, and support effective public health policy and hepatitis elimination efforts in Mauritania.

## Supplementary Information

Below is the link to the electronic supplementary material.

Supplementary file1 (PPTX 43 KB)

## Data Availability

All data extracted and used for this systematic review and meta-analysis are provided in the article and its Supplementary Materials.

## Abbreviations

HBV: Hepatitis B virus
DNA: Deoxyribonucleic acid
WHO: World Health Organization
HDV: Hepatitis D virus
HCC: Hepatocellular cellular carcinoma
LMICs: Low- and middle-income countries
NTDs: Neglected tropical diseases
PRISMA: Preferred Reporting Items for Systematic Reviews and Meta-Analyses
HBsAg: Hepatitis B surface antigen
HBeAg: Hepatitis B e antigen
anti-HDV: Anti-Hepatitis D Virus Antibodies
HIV: Human Immunodeficiency Virus
RNA: Ribonucleic Acid
MTCT: Mother-to-child transmission
PCR: Polymerase Chain Reaction
ELISA: Enzyme-Linked Immunosorbent Assay

## Declarations

### Statement of Generative AI in Scientific Writing

During the preparation of this work, the authors used ChatGPT to reformulate certain sentences. After using this tool, the authors reviewed and corrected the content where necessary and take full responsibility for the content of the publication.

## References

[1] Schaefer S. Hepatitis B virus taxonomy and hepatitis B virus genotypes. World J Gastroenterol. 2007;13:14–21. 10.3748/wjg.v13.i1.14. 17206751 10.3748/wjg.v13.i1.14 PMC4065870

[2] Hépatite B. 2024 [cited 2024 Dec 15]. https://www.who.int/fr/news-room/fact-sheets/detail/hepatitis-b. Accessed 15 Dec 2024.

[3] Si J, Yu C, Guo Y, Bian Z, Meng R, Yang L, et al. Chronic hepatitis B virus infection and total and cause-specific mortality: a prospective cohort study of 0.5 million people. BMJ Open. 2019;9(4):e027696. 10.1136/bmjopen-2018-027696. 30967410 10.1136/bmjopen-2018-027696 PMC6500223

[4] Bray F, Ferlay J, Soerjomataram I, Siegel RL, Torre LA, Jemal A. Global cancer statistics 2018: GLOBOCAN estimates of incidence and mortality worldwide for 36 cancers in 185 countries. CA Cancer J Clin. 2018;68:394–424. 10.3322/caac.21492. 30207593 10.3322/caac.21492

[5] Lampertico P, Degasperi E, Sandmann L, Wedemeyer H, Delta Cure 2022 Working Group. Hepatitis D virus infection: pathophysiology, epidemiology and treatment. Report from the first international delta cure meeting 2022. JHEP Rep. 2023;5:100818. 10.1016/j.jhepr.2023.100818. 37593170 10.1016/j.jhepr.2023.100818 PMC10428117

[6] Hepatitis D [Internet]. [cited 2025 May 12]. https://www.who.int/news-room/fact-sheets/detail/hepatitis-d. Accessed 12 May 2025.

[7] Cox AL, El-Sayed MH, Kao J-H, Lazarus JV, Lemoine M, Lok AS, et al. Progress towards elimination goals for viral hepatitis. Nat Rev Gastroenterol Hepatol. 2020;17:533–42. 10.1038/s41575-020-0332-6. 32704164 10.1038/s41575-020-0332-6 PMC7376316

[8] Polaris Elimination Maps – CDA Foundation [Internet]. 2024 [cited 2024 Dec 5]. https://cdafound.org/polaris/elimination-maps/. Accessed 5 Dec 2024.

[9] World Health Organization. Global hepatitis report 2017 [Internet]. Geneva: World Health Organization; 2017 [cited 2023 Aug 19]. https://apps.who.int/iris/handle/10665/255016. Accessed 19 Aug 2023.

[10] Depliant-RGPH5–1.pdf [Internet]. [cited 2025 Apr 1]. https://ansade.mr/wp-content/uploads/2024/09/Depliant-RGPH5-1.pdf. Accessed 1 Apr 2025.

[11] Nouakchott Population 2025 [Internet]. [cited 2025 Mar 15]. https://worldpopulationreview.com/cities/mauritania/nouakchott. Accessed 15 Mar 2025.

[12] Lunel Fabiani F, El Bara A, Hamed CT, Le Guillou Guillemette H. Hépatites Delta en Afrique: particularités épidémiologiques et cliniques. Med Trop Sante Int. 2023;3:mtsi.v3i4.2023.430. 10.48327/mtsi.v3i4.2023.430. 38390020 10.48327/mtsi.v3i4.2023.430 PMC10879896

[13] Page MJ, McKenzie JE, Bossuyt PM, Boutron I, Hoffmann TC, Mulrow CD, et al. The PRISMA 2020 statement: an updated guideline for reporting systematic reviews. BMJ. 2021;372:n71. 10.1136/bmj.n71. 33782057 10.1136/bmj.n71 PMC8005924

[14] Checklist_for_Prevalence_Studies.pdf [Internet]. [cited 2025 Mar 17]. https://jbi.global/sites/default/files/2020-08/Checklist_for_Prevalence_Studies.pdf. Accessed 17 Mar 2025.

[15] Chukiat Viwatwongkasem, Khanokporn Donjdee, Tantanut Poodphraw. Profile Likelihood Tests for Common Risk Ratios in Meta-Analysis Studies [Internet]. Open J. Stat. 2018. https://www.scirp.org/reference/referencespapers?referenceid=2412693.

[16] Rui WZ, Baïdy BL, N’Diaye M. Étude de l’infection du virus de l’hépatite B en milieu scolaire de Kiffa et Sélibaby, Mauritanie. 9773202

[17] El Bara A, Pivert A, Veillon P, Ng Wing Sang C, Bollahi M, Abdel K, et al. From national HBV and HDV screenings to vaccination and treatment in healthcare workers: the Mauritanian pilot study. Vaccine. 2021;39:2274–9. 10.1016/j.vaccine.2021.03.011. 33752951 10.1016/j.vaccine.2021.03.011

[18] Malkin JE, Druilhe P, Couroucé AM, Monjour L, Gentilini M. Distribution géographique et ethnique des sous-types d’antigènes HBs en Mauritanie. Rev Fr Transfus Immuno-Hématologie. 1983;26:591–7. 10.1016/S0338-4535(83)80074-9. 10.1016/s0338-4535(83)80074-9 6675158

[19] Boushab BM, Mohamed Limame OCM, Fatim Zahra F-M, Mamoudou S, Roseline Darnycka BM, Saliou SM. Estimation of seroprevalence of HIV, hepatitis B and C virus and syphilis among blood donors in the hospital of Aïoun, Mauritania. Pan Afr Med J. 2017;28:118. 10.11604/pamj.2017.28.118.12465 10.11604/pamj.2017.28.118.12465 PMC5837177 29515736

[20] Mohamed Lemine Ould Salem, Moctar Elbou Bellamech. Prevalence and Clinico-Biological Characteristics of Viral Hepatitis B and C in Chronic Hemodialysis Patients at the National Hospital Center of Nouakchott-Mauritania [Internet]. Auctores. [cited 2024 Nov 21]. https://www.auctoresonline.org/article/prevalence-and-clinico-biological-characteristics-of-viral-hepatitis-b-and-c-in-chronic-hemodialysis-patients-at-the-national-hospital-center-of-nouakchott-mauritania. Accessed 21 Nov 2024.

[21] Mansour W, Malick F-ZF, Sidiya A, Ishagh E, Chekaraou MA, Veillon P, et al. Prevalence, risk factors, and molecular epidemiology of hepatitis B and hepatitis delta virus in pregnant women and in patients in Mauritania. J Med Virol [Internet]. 2012 [cited 2023 July 12];84:1186–98. 10.1002/jmv.23336. 10.1002/jmv.23336 22711346

[22] Mansour W, Bollahi M-A, Hamed C-T, Brichler S, Le Gal F, Ducancelle A, et al. Virological and epidemiological features of hepatitis delta infection among blood donors in Nouakchott, Mauritania. J Clin Virol. 2012;55:12–6. 10.1016/j.jcv.2012.05.011. 22704272 10.1016/j.jcv.2012.05.011

[23] Lo BB, Meymouna M, Boulahi MA, Tew M, Sow A, Ba A, et al. Prevalence of serum markers of hepatitis B and C virus in blood donors of Nouakchott, Mauritania. Bull Soc Pathol Exot. 1990;1999(92):83–4. 10399594

[24] Lunel-Fabiani F, Mansour W, Amar AO, Aye M, Le Gal F, Malick F-ZF, et al. Impact of hepatitis B and delta virus co-infection on liver disease in Mauritania: a cross sectional study. J Infect. 2013;67:448–57. 10.1016/j.jinf.2013.06.008. 10.1016/j.jinf.2013.06.008 23796871

[25] Le Guillou-Guillemette H, Pivert A, ElBara A, Vall M, Sang CNW, Veillon P, et al. Prevalence, clinical and virological characteristics and short-term prognosis of hepatitis delta infection among patients with HIV/HBV in Nouakchott, Mauritania. J Viral Hepat [Internet]. 2024 [cited 2024 June 7];n/a. 10.1111/jvh.13950. 10.1111/jvh.13950 38771311

[26] WHO Immunization Data portal - Detail Page [Internet]. Immun. Data. [cited 2024 Nov 25]. https://immunizationdata.who.int/global/wiise-detail-page. Accessed 25 Nov 2024.

[27] Asrani SK, Devarbhavi H, Eaton J, Kamath PS. Burden of liver diseases in the world. J Hepatol. 2019;70:151–71. 10.1016/j.jhep.2018.09.014. 30266282 10.1016/j.jhep.2018.09.014

[28] Stockdale AJ, Kreuels B, Henrion MYR, Giorgi E, Kyomuhangi I, Martel C, et al. The global prevalence of hepatitis D virus infection: systematic review and meta-analysis. J Hepatol. 2020;73(3):523–32. 10.1016/j.jhep.2020.04.008. 32335166 10.1016/j.jhep.2020.04.008 PMC7438974

[29] Feindiri M, Kabbaj H, Salihoun M, Errabih I, Melloul M, Elfahime E, et al. Hepatitis B surface antigen seroprevalence in Morocco (2000–2024): a systematic review and meta-analysis. BMC Infect Dis. 2025;25:1607. 10.1186/s12879-025-12002-1. 41254550 10.1186/s12879-025-12002-1 PMC12625385

[30] Masson E. Hépatite B en Tunisie. Épidémiologie, facteurs de risque et impact de la vaccination [Internet]. EM-Consulte. [cited 2025 Dec 23]. https://www.em-consulte.com/article/1288529/hepatite-b-en-tunisie-epidemiologie-facteurs-de-ri. Accessed 23 Dec 2025.

[31] Ezzikouri S, Pineau P, Benjelloun S. Hepatitis B virus in the Maghreb Region: from epidemiology to prospective research. Liver Int. 2013;33:811–9. 10.1111/liv.12135. 23530901 10.1111/liv.12135

[32] Stockdale AJ, Chaponda M, Beloukas A, Phillips RO, Matthews PC, Papadimitropoulos A, et al. Prevalence of hepatitis D virus infection in sub-Saharan Africa: a systematic review and meta-analysis. Lancet Glob Health. 2017;5:e992-1003. 10.1016/S2214-109X(17)30298-X. 28911765 10.1016/S2214-109X(17)30298-X PMC5599428

[33] Wembulua BS, Le Gal F, Ndiaye O, Pandi MS, Akotia MK, Badiane AS, et al. Hepatitis Delta and liver disease among people living with hepatitis B with or without HIV co‐infection in Senegal. Liver Int. 2025;45(3):e70026. 10.1111/liv.70026. 39967446 10.1111/liv.70026 PMC11836594

[34] Marino Q, Cisse ,Moussa, Gerber ,Athenaïs, Dolo ,Oumar, Sayon ,Sophie, Ba ,Alhassane, et al. Low hepatitis D seroprevalence in blood donors of Bamako, Mali. Infect Dis [Internet]. Taylor & Francis; 2019 [cited 2025 Apr 24];51:622–4. 10.1080/23744235.2019.1620963. 10.1080/23744235.2019.1620963 31165648

[35] Jaquet A, Wandeler G, Tine J, Diallo MB, Manga NM, Dia NM, et al. Prevention and care of hepatitis B in Senegal; awareness and attitudes of medical practitioners. Am J Trop Med Hyg. 2017;97:389–95. 10.4269/ajtmh.17-0065. 28829726 10.4269/ajtmh.17-0065 PMC5544102

[36] Konaté A, Coulibaly HSW, Samaké KDW, Dicko MY, Dakouo RDW, Kaya ASW, et al. Epidemiological and Serological Profile of Hepatitis B Virus in an Urban Area in Mali. Open J Gastroenterol [Internet]. Scientific Research Publishing; 2019 [cited 2025 Apr 24];9:158–63. 10.4236/ojgas.2019.98018.

[37] Queiroz JAdaS, Arruda MB, Roca TP, Araújo A, Passos-Silva AM, de Castro e Silva E, et al. Evaluation of HDV stability under different biological sample collection and storage conditions: implications for molecular diagnosis. Virol J. 2025;22(1):383. 10.1186/s12985-025-02919-z. 41272652 10.1186/s12985-025-02919-z PMC12639762

[38] Easterbrook PJ, Luhmann N, Bajis S, Min MS, Newman M, Lesi O, et al. WHO 2024 hepatitis B guidelines: an opportunity to transform care. The Lancet Gastroenterology & Hepatology. 2024;9:493–5. 10.1016/S2468-1253(24)00089-X. 38614110 10.1016/S2468-1253(24)00089-X

[39] Drucker E, Alcabes PG, Marx PA. The injection century: massive unsterile injections and the emergence of human pathogens. Lancet. 2001;358:1989–92. 10.1016/S0140-6736(01)06967-7. 11747942 10.1016/S0140-6736(01)06967-7

[40] Gall D. The chemoprophylaxis of sleeping sickness with the diamidines. Ann Trop Med Parasitol. 1954;48:242–58. 10.1080/00034983.1954.11685622. 13208153 10.1080/00034983.1954.11685622

[41] La pandémie de COVID-19 à l’origine du plus grand recul ininterrompu des vaccinations en trente ans [Internet]. [cited 2025 Dec 23]. https://www.who.int/fr/news/item/15-07-2022-covid-19-pandemic-fuels-largest-continued-backslide-in-vaccinations-in-three-decades. Accessed 23 Dec 2025.

[42] El Hachimi H, El Alem MMM, Haimoudane E, Yebouk C, Pedersen J, Fall-Malick F-Z, et al. The first assessments of pediatric HBV immunization coverage in Mauritania and persistence of antibody titers post infant immunizations. Vaccines. 2023;11(3):588. 10.3390/vaccines11030588. 36992174 10.3390/vaccines11030588 PMC10059282

[43] Hiebert-Suwondo L, Manning J, Tohme RA, Buti M, Kondili LA, Spearman CW, et al. A 2024 global report on national policy, programmes, and progress towards hepatitis B elimination: findings from 33 hepatitis elimination profiles. Lancet Gastroenterol Hepatol. 2025;10:671–84. 10.1016/S2468-1253(25)00069-X. 40409324 10.1016/S2468-1253(25)00069-X PMC12308976

[44] Su F-H, Chen J-D, Cheng S-H, Lin C-H, Liu Y-H, Chu F-Y. Seroprevalence of hepatitis-B infection amongst Taiwanese university students 18 years following the commencement of a national hepatitis-B vaccination program. J Med Virol. 2007;79(2):138–43. 10.1002/jmv.20771. 17177303 10.1002/jmv.20771

