# Supplementary material for: Seroprevalence and Molecular Epidemiology of Hepatitis B and D Viruses in Mauritania: a Systematic Review and Meta-Analysis
Source: J Epidemiol Glob Health. 2026 Apr 17;16(1):65. doi: 10.1007/s44197-026-00559-2 (PMC13222904; doi:10.1007/s44197-026-00559-2)
Supplement: Supplementary file 1 — Supplementary file1 (PPTX 43 KB) [file 44197_2026_559_MOESM1_ESM.pptx]

## Slide 1
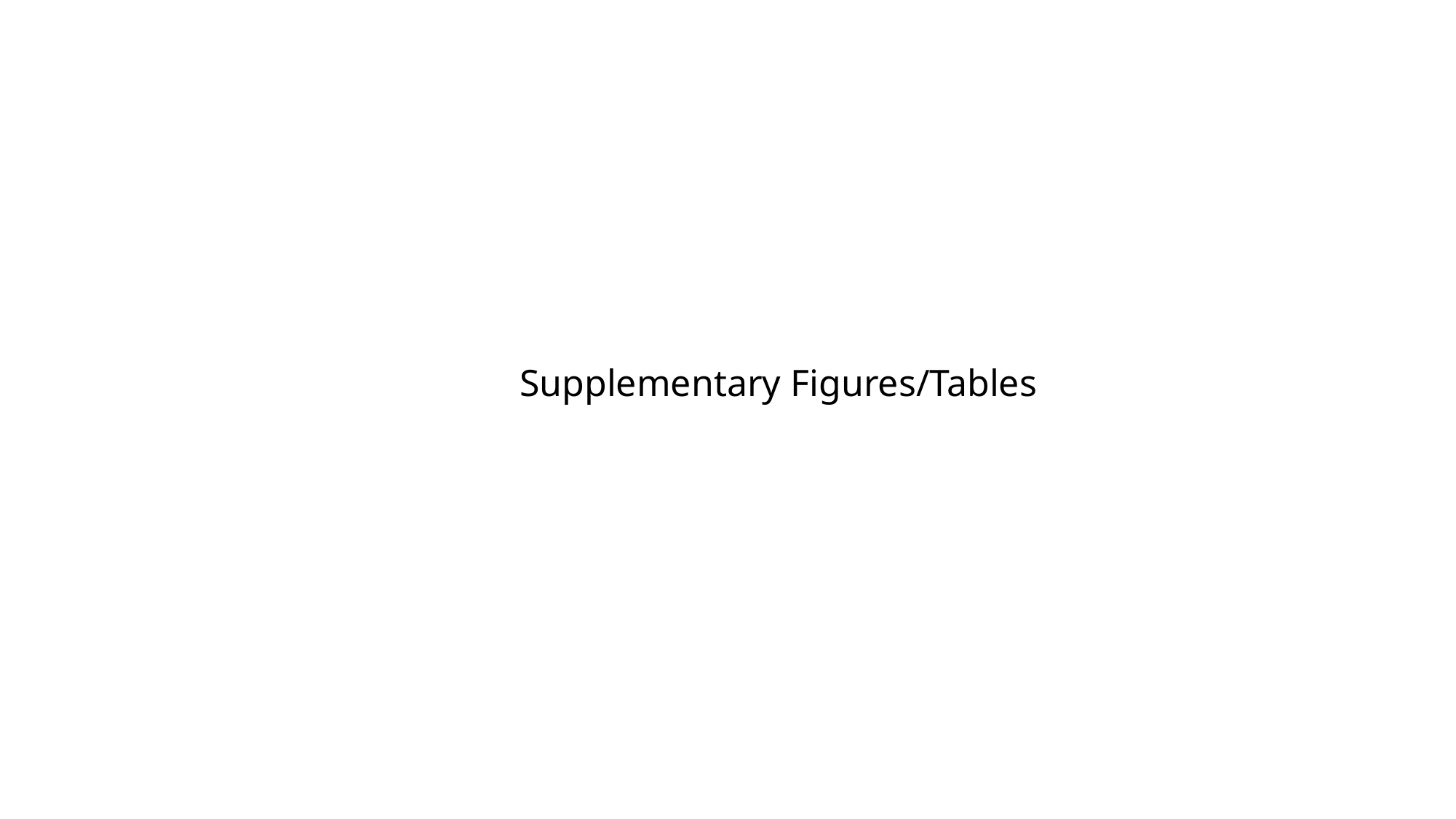

Supplementary Figures/Tables

## Slide 2
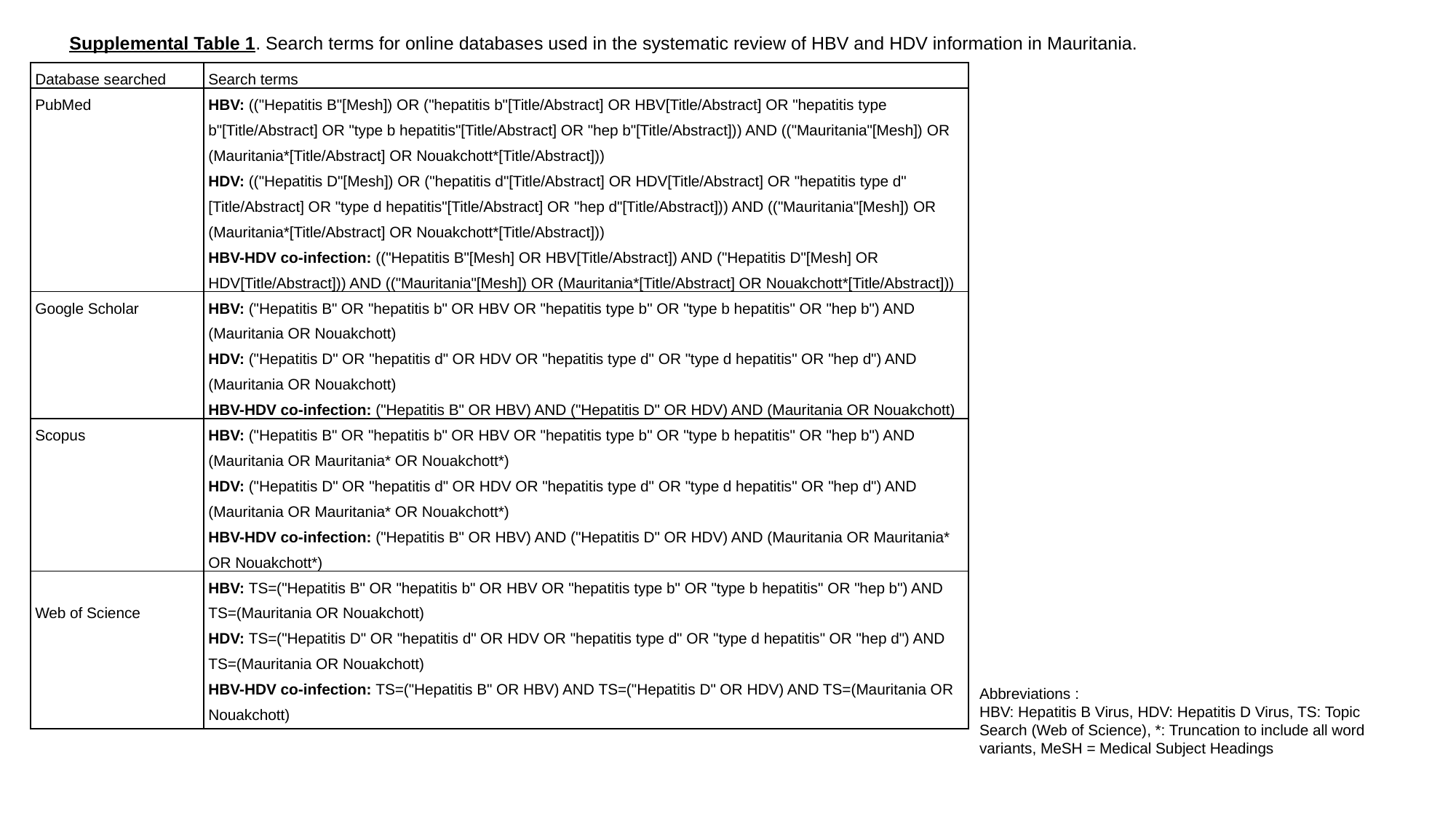

Supplemental Table 1. Search terms for online databases used in the systematic review of HBV and HDV information in Mauritania.
| Database searched | Search terms |
| --- | --- |
| PubMed | HBV: (("Hepatitis B"[Mesh]) OR ("hepatitis b"[Title/Abstract] OR HBV[Title/Abstract] OR "hepatitis type b"[Title/Abstract] OR "type b hepatitis"[Title/Abstract] OR "hep b"[Title/Abstract])) AND (("Mauritania"[Mesh]) OR (Mauritania\*[Title/Abstract] OR Nouakchott\*[Title/Abstract])) HDV: (("Hepatitis D"[Mesh]) OR ("hepatitis d"[Title/Abstract] OR HDV[Title/Abstract] OR "hepatitis type d" [Title/Abstract] OR "type d hepatitis"[Title/Abstract] OR "hep d"[Title/Abstract])) AND (("Mauritania"[Mesh]) OR (Mauritania\*[Title/Abstract] OR Nouakchott\*[Title/Abstract])) HBV-HDV co-infection: (("Hepatitis B"[Mesh] OR HBV[Title/Abstract]) AND ("Hepatitis D"[Mesh] OR HDV[Title/Abstract])) AND (("Mauritania"[Mesh]) OR (Mauritania\*[Title/Abstract] OR Nouakchott\*[Title/Abstract])) |
| Google Scholar | HBV: ("Hepatitis B" OR "hepatitis b" OR HBV OR "hepatitis type b" OR "type b hepatitis" OR "hep b") AND (Mauritania OR Nouakchott) HDV: ("Hepatitis D" OR "hepatitis d" OR HDV OR "hepatitis type d" OR "type d hepatitis" OR "hep d") AND (Mauritania OR Nouakchott) HBV-HDV co-infection: ("Hepatitis B" OR HBV) AND ("Hepatitis D" OR HDV) AND (Mauritania OR Nouakchott) |
| Scopus | HBV: ("Hepatitis B" OR "hepatitis b" OR HBV OR "hepatitis type b" OR "type b hepatitis" OR "hep b") AND (Mauritania OR Mauritania\* OR Nouakchott\*) HDV: ("Hepatitis D" OR "hepatitis d" OR HDV OR "hepatitis type d" OR "type d hepatitis" OR "hep d") AND (Mauritania OR Mauritania\* OR Nouakchott\*) HBV-HDV co-infection: ("Hepatitis B" OR HBV) AND ("Hepatitis D" OR HDV) AND (Mauritania OR Mauritania\* OR Nouakchott\*) |
| Web of Science | HBV: TS=("Hepatitis B" OR "hepatitis b" OR HBV OR "hepatitis type b" OR "type b hepatitis" OR "hep b") AND TS=(Mauritania OR Nouakchott) HDV: TS=("Hepatitis D" OR "hepatitis d" OR HDV OR "hepatitis type d" OR "type d hepatitis" OR "hep d") AND TS=(Mauritania OR Nouakchott) HBV-HDV co-infection: TS=("Hepatitis B" OR HBV) AND TS=("Hepatitis D" OR HDV) AND TS=(Mauritania OR Nouakchott) |
Abbreviations :
HBV: Hepatitis B Virus, HDV: Hepatitis D Virus, TS: Topic Search (Web of Science), *: Truncation to include all word variants, MeSH = Medical Subject Headings

## Slide 3
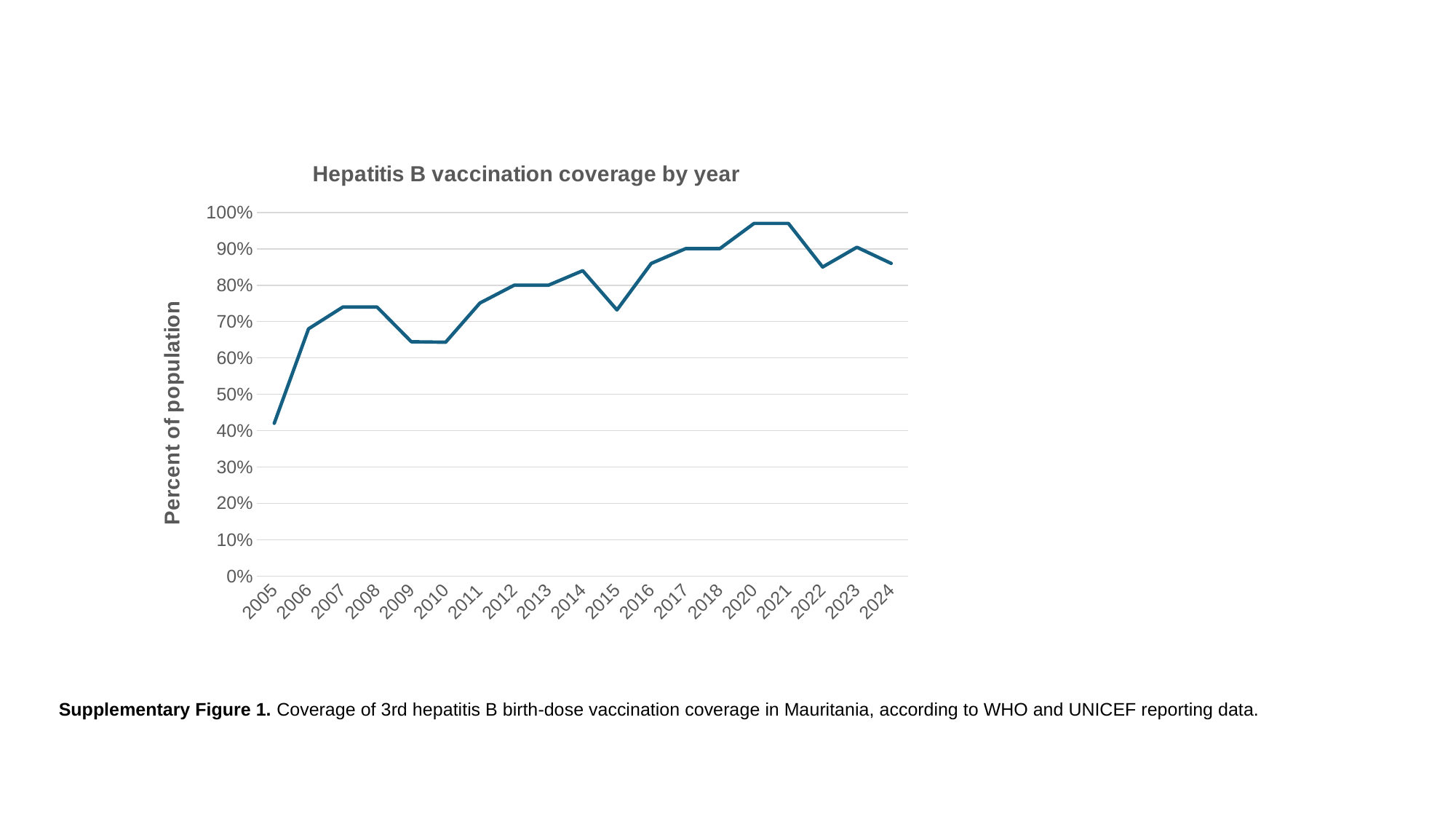

### Chart: Hepatitis B vaccination coverage by year
| Category | Hepatitis B vaccination coverage by years |
|---|---|
| 2005 | 0.42 |
| 2006 | 0.68 |
| 2007 | 0.74 |
| 2008 | 0.74 |
| 2009 | 0.6445 |
| 2010 | 0.6432 |
| 2011 | 0.751 |
| 2012 | 0.8 |
| 2013 | 0.8 |
| 2014 | 0.84 |
| 2015 | 0.732 |
| 2016 | 0.86 |
| 2017 | 0.9005 |
| 2018 | 0.9005 |
| 2020 | 0.97 |
| 2021 | 0.97 |
| 2022 | 0.85 |
| 2023 | 0.9044 |
| 2024 | 0.86 |Supplementary Figure 1. Coverage of 3rd hepatitis B birth-dose vaccination coverage in Mauritania, according to WHO and UNICEF reporting data.
